# Proportional Topology Optimization: A New Non-Sensitivity Method for Solving Stress Constrained and Minimum Compliance Problems and Its Implementation in MATLAB

**DOI:** 10.1371/journal.pone.0145041

**Published:** 2015-12-17

**Authors:** Emre Biyikli, Albert C. To

**Affiliations:** Department of Mechanical Engineering and Materials Science & Center for Simulation and Modeling, University of Pittsburgh, Pittsburgh, Pennsylvania, United States of America; Nankai University, CHINA

## Abstract

A new topology optimization method called the Proportional Topology Optimization (PTO) is presented. As a non-sensitivity method, PTO is simple to understand, easy to implement, and is also efficient and accurate at the same time. It is implemented into two MATLAB programs to solve the stress constrained and minimum compliance problems. Descriptions of the algorithm and computer programs are provided in detail. The method is applied to solve three numerical examples for both types of problems. The method shows comparable efficiency and accuracy with an existing optimality criteria method which computes sensitivities. Also, the PTO stress constrained algorithm and minimum compliance algorithm are compared by feeding output from one algorithm to the other in an alternative manner, where the former yields lower maximum stress and volume fraction but higher compliance compared to the latter. Advantages and disadvantages of the proposed method and future works are discussed. The computer programs are self-contained and publicly shared in the website www.ptomethod.org.

## Introduction

Topology optimization can be regarded as the systematic removal of redundant material from the design domain in order to attain design with higher strength-to-weight ratios. It is getting an increasing amount of attention since its introduction to truss structures by Michell [1] and continuum structures by Bendsoe and Kikuchi [2]. Even further, recently popular additive manufacturing techniques appreciate the importance of topology optimization since it facilitates the manufacture of porous structural designs with much complicated geometries.

Topology optimization methods are required to provide designers with black-and-white (or 1/0) designs to easily identify structural members as black regions and voided regions as white regions. On the contrary, it was noticed that topology optimization methods with continuous design variables are more successful for minimization of the objective function [3]. For this reason, continuum design variables with penalization methods are highly favored, such as the Solid Isotropic Material with Penalization (SIMP) introduced by Bendsoe [4]. It is very important to realize that, discrete or continuous, topology optimization is only a conceptual tool and requires post-processing of the optimized geometry. Two popular design problems are the stress constrained problem, which aims at minimizing volume fraction while satisfying stress constraints and the minimum compliance problem, which aims at minimizing compliance for a given volume fraction. In short, these problems will be referred to as stress problem and compliance problem hereafter. Also, the word “element” always refers to the finite element (FE) of an FE mesh in this work. In this context, the design variable can be imagined as thickness of a plate [5] or scaling factor of a unit cell in a cellular structure [6].

The compliance problem has been widely investigated by Bendsoe and Sigmund [5], Sigmund [7], and Stolpe and Svanberg [8], to name a few. Open source computer programs to solve this type of problem are distributed [7, 9–13]. On the other hand, it is a well-known fact that stress analysis is a more significant concern for designers. Compared to compliance problems, however, stress problems bear more challenging difficulties such as high non-linearity [14]. The stress problem and related issues has been studied by Lee [15], Duysinx and Bendsoe [16], and Paris et al. [17] to name a few. Nevertheless, probably due to its added commercial value and complexity, there is no open source distribution of such a computer program for continua.

Numerous topology optimization techniques have been developed to solve both types of problems. Among these are mathematical methods such as optimality criteria method, convex linearization method, method of moving asymptotes, successive linear programming, and evolutionary structural optimization method; and stochastic methods such as simulated annealing, harmony search algorithm, immune algorithm, differential evolution, and genetic algorithm. For a broader list of methods, see Sigmund [18] and Rozvany [19].

The Optimality Criteria (OC) method is the most fundamental as compared to the other listed mathematical methods [20] and was first introduced in structural design by Prager [21]. The method assigns design variables to elements proportionally to the values of the objective function [22]. In this respect, it is an efficient and simple method. Sigmund et al. [7] employs the OC method in the TOP99 computer program, which is a 99-line MATLAB code that solves the compliance problem for the Messerschmitt-Bölkow-Blohm (MBB) beam.

The Successive Linear Programming (SLP) method linearizes the originally nonlinear problem at a design point and then locally optimizes the linear problem within a region bounded by some move limits. The local optimization problem can be solved by, for instance, the simplex algorithm [23]. The SQP method is only different from the SLP method in converting the originally nonlinear problem into a quadratic problem. As opposed to SLP, the Convex Linearization (CONLIN) method performs linearization with different variables with respect to the characteristics of the optimization problem [24]. In this respect, the Method of Moving Asymptotes (MMA) is a specific version of CONLIN in that the search behavior is more aggressively controlled by moving limits [25]. The reader is referred to the book by Christensen [26] for further details on these methods.

The Evolutionary Structural Optimization (ESO) method starts with a full design domain and then iteratively removes elements from the domain with respect to the values of the objective function [27, 28]. If the method also includes addition of elements, it is then called Bidirectional ESO (BESO). Addition/removal of elements render a discreteness that is earlier noted as a bad attribute in terms of minimization performance. Also, it is argued that the ESO solutions are highly mesh dependent [29] and not guaranteed to reach the optimal design [30]. Indeed, ESO resembles the Fully Stressed Design (FSD) method, which dictates removal of material from an element until the element is fully stressed [31]. FSD may also be considered a simple OC method. Its performance to yield an optimal solution is questioned by Rozvany [19].

Stochastic methods utilize randomness in order to imitate rules of the nature. Although continuous versions are viable [32], stochastic methods are usually discrete thus they are also referred to as combinatorial optimization methods or population based methods. Simulated annealing is the process of searching for the best configuration by a statistical analysis of the cost distribution. In each iteration, the method draws a random configuration that is unconditionally accepted if it has a lower cost or conditionally accepted depending on an acceptance rate otherwise. The acceptance rate is gradually driven to zero during progress of the simulation. The method is favored to be easily controlled due to only one control parameter [33]. Evolutionary algorithms represent a general framework for many other specialized methods [34]. The method retains a set of solutions and produces a new one in every iteration by the following three operators: reproduction, crossover, and mutation. Reproduction promotes the most favorable solutions to imitate the survival of the fittest; crossover crosses two solutions in order to create a new solution imitating the crossbreeding; and, mutation introduces new random elements into solutions to increase the diversity of the set. Harmony search algorithm is inspired from the musical process of naturally searching for a perfect state of harmony. In each iteration, the method creates a new solution by randomly blending elements from solutions of the set or from the allowed range in order to replace the worst solution of the set. Harmony search algorithm is favored for bringing together unique features of other stochastic methods such as history dependence of tabu search, varying adaptation rate of simulated annealing, and combination of a set of solutions of genetic algorithms [35]. Immune algorithms imitate concepts and use terminology of the natural biological immune systems such as antigen (objective), antibody (chromosome/solution), and affinity (fitness). In their method, called the Multi-Modal Immune Algorithm (MMIA), Luh et al restore the imbalance of evolutionary algorithms between exploitation (reproduction) and exploration (crossover and mutation) mechanisms in order to eliminate local search inability and premature convergence of evolutionary algorithms [29]. Different from common evolutionary algorithms, Differential Evolution (DE) method utilizes a weighted difference of two or more solutions in order to get a mutated solution. Wu et al. emphasizes the binary representation in DE methods by developing the Modified Binary Differential Evolution (MBDE) method [30] where they introduce a novel binary mutation operator that is particularly ensured to produce equal amounts of 0 and 1. Finally, there are more stochastic methods in the literature: rule-based optimization by Russel and Manoochehri [36], ant algorithm [37], and tabu search [38].

Advantages and disadvantages of the stochastic methods are discussed in many ways. One of the most pronounced advantages is that they perform a global search whereas mathematical methods are stuck to a local minimum [35]. For this reason, they do not require a good starting point, are independent of the starting point, and are better at exploring the design landscape [30]. In this regard, they do not have difficulties with multiple or sharp peaks of the objective function and constraints [35]. They need fewer mathematical requirements such as calculation of gradients. Also, they directly solve 0/1 problems without a need for penalization, post-processing, or filtering. On the contrary, the most pronounced disadvantage of stochastic methods is the large number of design combinations, which hinders the effectiveness of the search process. Also, the cost of function evaluations and checking for connectivity in every iteration may become highly costly due to convergence in large number of iterations [33].

Among the introduced methods, OC, SLP, SQP, CONLIN, and MMA require calculation of the sensitivities of objective function and constraints. In the TOP99 MATLAB code, the sensitivity of compliance is calculated by taking derivative of the SIMP expression with respect to density [7]. On the contrary, more rigorous sensitivity calculations are usually employed in stress problems [39, 40]. These sensitivities, especially for stress, are analytically complicated to derive and their computation brings an additional computational burden. For instance, it is difficult to obtain sensitivities without simplification of the derivation for more complex topology optimization problems such as compliant mechanism synthesis using nonlinear analysis and crashworthiness design using dynamic analysis [41]. It is also reported that sensitivity analysis is complicated due to change of the interface between structure and acoustic medium in an exterior acoustic problem [42]. Besides, computation of sensitivities may introduce some implementation concerns. For example, computation of the sensitivity analysis may become infeasible in practical applications because of large number of stress constraints imposed [40]. In another example, it is argued that sensitivity analyses are computationally intensive in acoustic problems since both structural and acoustic analyses are carried out in every iteration [43]. Despite its additional computational load and implementation complexity, sensitivity information is useful in optimization. For a similar discussion on gradient versus non-gradient methods, the reader is referred to the forum article by Sigmund (2011). As a conclusion, there is a trade-off between sensitivity and non-sensitivity methods in terms of computational/implementation complexity and efficiency.

In this paper, a simple and efficient non-sensitivity method, called the Proportional Topology Optimization (PTO), is presented to perform topology optimization for stress (PTOs) and compliance (PTOc) problems. The PTO algorithm assigns the design variables to elements proportionally to the value of stress in the stress problem and compliance in the compliance problem. In particular, it imposes constraints only globally on the entire system. Accordingly, it globally manages the proportional distribution of design variables to the elements. This global approach substantially differentiates PTO from FSD since the latter employs an element-wise approach. It is admitted that PTO method is highly heuristic and searches for the optimized solutions. Nevertheless, it is this heuristic that makes the method simple to understand and implement. Also, the method does not incorporate sensitivities; therefore, it avoids the complications associated with sensitivities. Employment of continuous density variables improves the search performance of the method and preserves the flexibility to design for intermediate densities. Results indicate that the method produces efficient and accurate solutions in consideration of its simplicity. It is important to note that although PTO is highly heuristic, its performance is much better than stochastic methods as will be shown by its comparison of its efficiency to mathematical methods later.

Inspired by the TOP99 computer program, the method is implemented into two MATLAB programs individually for the stress and compliance problems that solve the MBB beam example. The computer programs are implemented as self-contained MATLAB functions such that they do not even depend on optional MATLAB toolboxes. The authors are distributing the source of computer programs freely for educational and research purposes in the website www.ptomethod.org. To the best of the authors’ knowledge, PTOs is the first publicly shared and self-contained computer program that solves the stress constrained problem for continua.

The paper presents, in order, stress and compliance problems, the PTO algorithms, numerical examples, and conclusions. Computer programs are in S1 and S2 Appendices.

## Stress and compliance problems

Two types of problems, i.e., stress constrained problem and minimum compliance problem, are issued in the following sections.

### 2.1. Stress constrained problem

The stress problem is the minimization of volume fraction while satisfying the stress constraints. The optimization problem reads

$$\{\begin{array}{c} \text{min}\sum_{i}^{N}\rho_{i}v_{i}\\ such\;that\;\{\begin{array}{c} \mathrm{Ku}=f\\ \sigma_{i}\leq \sigma_{l}\;if\;\rho >0\\ 0\leq \rho_{min}\leq \rho_{i}\leq \rho_{max}\leq 1 \end{array} \end{array}$$ (1)

where *N* is the number of elements, *ρ* is the density (and also the design variable), *ρ*_*i*_ is the elemental density, *v*_*i*_ is the elemental area/volume, ***K*** is the stiffness matrix, ***u*** is the displacement vector, ***f*** is the external force vector, *σ*_*i*_ is the elemental stress measure (e.g., von Mises), *σ*_*l*_ is the stress limit, *ρ*_*min*_ is the lower bound on elemental density, and *ρ*_*max*_ is the upper bound on elemental density. Typically, *ρ* is limited to [*ρ*_*min*_, 1] where *ρ*_*min*_ is 0.001 [17] to preclude stiffness singularities [44]. Although the problem is posed as minimization of the total mass, it is usually referred to as minimization of the volume fraction for practical reasons. Minimization of these terms is equivalent from the optimization point of view. A volume fraction 0 means void while 1 means solid element. The stress problem is noted to be non-convex and highly non-linear [17].

### 2.2. Minimum compliance problem

The compliance problem is minimization of the compliance while satisfying the volume fraction constraint. The optimization problem reads

$$\{\begin{array}{c} \text{min}\;C=u^{T}\mathrm{Ku}\\ such\;that\;\{\begin{array}{c} \mathrm{Ku}=f\\ \sum_{i}^{N}\rho_{i}v_{i}=M\\ 0\leq \rho_{min}\leq \rho_{i}\leq \rho_{max}\leq 1 \end{array} \end{array}$$ (2)

where, in addition to the nomenclature given for the stress problem, *C* is the compliance and *M* is the total mass.

## The PTO Algorithms

Algorithms of the PTO method to solve the stress (PTOs) and compliance (PTOc) problems are described in the following.

Table 1 presents the PTOs algorithm. The algorithm starts with setup of vectors and matrices for FE and stress analyses and filtering. Then, the algorithm goes into the main loop. Every iteration of the main loop starts with FE and stress analyses. Following, the termination criteria is checked. That is, whether the maximum elemental stress in the system is close to the allowable stress limit within a prescribed tolerance, which is set equal to 0.001 in this work. If the criterion returns true, the simulation terminates. Otherwise, the algorithm continues to optimize the topology. The first step of optimization part is to determine the target material amount, which is going to be the new material amount in the system. In other words, the current material amount will be updated to the target material amount. If the maximum elemental stress in the system is bigger than the allowable stress limit, then the current material amount is increased by a material move amount. Otherwise, the current material amount is decreased by the same material move amount. The material move amount scales with the number of elements (0.001 x *number of elements*) and is kept constant during the course of the simulation. In the next step, the algorithm distributes the target material amount to the elements. The target material amount can only be distributed iteratively for the reasons that will be explained in the following. Because of this iterative procedure, the material amount to be distributed is called the remaining material amount, and the iterative procedure initiates with a remaining material amount that is equal to the target material amount.

**Table 1:** PTOs algorithm to solve the stress problem.

Algorithm				
Setup FE and stress analyses and filtering				
Until convergence				
	Perform FE and stress analyses			
	Check stop criteria, break if satisfied			
	Run optimization algorithm			
		Determine TM		
			Distribute RM	
				If stress limit is exceeded, TM = CM + MM
				Else, TM = CM—MM
		Set RM = TM		
		Until RM is small enough		
			Distribute RM to elements proportionally to their stress values	
			Apply filter	
			Apply density limits	
			Calculate AM	
			Update RM = TM–AM	
		Update density		

where TM is the target material amount, CM is the current material amount, MM is the material move amount, RM is the remaining material amount, and AM is the actual material amount.

In order to perform the iterative distribution of the target material amount, the algorithm goes into an inner loop. The distribution is conducted proportionally to the elemental stress values. The degree of proportion is extended to the power of *q* such that

$$\rho_{i}^{opt}=\frac{RM}{\sum_{j}^{N}\sigma_{j}^{q}v_{j}}\sigma_{i}^{q}$$ (3)

where *RM* is the remaining material amount, *N* is the number of elements, *ρ*_*i*_^*opt*^ is the optimized elemental density, *σ*_*i*_ is the elemental stress measure, *v*_*j*_ is the elemental volume, and *q* is the proportion exponent (will be detailed in section 3.7). Apparently, the above relation distributes the remaining material amount regardless of density limits. The enforcement of density limits on the elements trims the distributed material amount to the lower and upper bounds if the bounds are exceeded. As a result, the actual material amount is different than the target material amount. This difference is the reason for distributing the remaining material amount iteratively in an inner loop until the target material amount is reached. Every iteration of the inner loop starts with distributing the remaining material amount. It is followed by application of filtering and density limits. In this work, a volume preserving density filtering is used, which will be explained in detail later. At the end of the inner loop, the actual material amount, which is left after enforcing limits and filtering, is calculated. The remaining material amount is then the actual material amount subtracted from the target material amount. In the next iteration of inner loop, this remaining material amount is redistributed following the same routine. The inner loop runs until the remaining material amount is small enough.

The final step of main loop updates the elemental densities by linearly blending elemental densities from the previous iteration and optimized elemental densities in the current iteration. The update scheme reads

$$\rho_{i}^{new}=\alpha \rho_{i}^{prev}+\left(1-\alpha \right)\rho_{i}^{opt}$$ (4)

where *ρ*_*i*_ is the elemental density, *ρ* ^*new*^ is the new elemental density to be passed to the next iteration, *ρ* ^*prev*^ is the elemental density from the previous iteration, *ρ* ^*opt*^ is the optimized elemental density in the current iteration, and *α* is the history coefficient (will be detailed in section 3.7). The history coefficient decides the ratios of elemental densities from both sides, and it is unitless. For instance, a value of 0 eliminates elemental density from the previous iteration and indicates no dependence on the history.

PTOc algorithm (Table 2) is slightly different from the PTOs algorithm. The most prominent difference is the determination of the target material amount. PTOc algorithm does not need to modify the target material amount since it is constrained to a fixed amount by the definition of the problem. For this reason, PTOc algorithm calculates the target material amount once at the beginning of the simulation and uses it thereafter. Another difference is the distribution of the target material amount. PTOc distributes the target material amount proportionally to the elemental compliance values instead of the elemental stress values. The distribution equation then reads

$$\rho_{i}^{opt}=\frac{RM}{\sum_{j}^{N}C_{j}^{q}v_{j}}C_{i}^{q}$$ (5)

where *RM* is the remaining material amount, *N* is the number of elements, *ρ*_*i*_^*opt*^ is the optimized elemental density, *C*_*i*_ is the elemental compliance value, *v*_*j*_ is the elemental volume, and *q* is the proportion exponent. The elemental compliance values are recalculated in every iteration at the beginning of the main loop. The last difference is the termination criterion of the main loop. The main loop stops if the maximum change in elemental densities between two successive iterations is smaller than a prescribed tolerance, which is equal to 0.01 in this work. The rest of the steps are identical to the PTOs algorithm.

**Table 2:** PTOc algorithm to solve the compliance problem.

Algorithm			
Setup FE and compliance analyses and filtering			
Determine TM			
Until convergence			
	Perform FE and compliance analyses		
	Check stop criteria, break if satisfied		
	Run optimization algorithm		
		Set RM = TM	
		Until RM is small enough	
			Distribute RM to elements proportionally to their compliance values
			Apply filter
			Apply density limits
			Calculate AM
			Update RM = TM–AM
			Update density

where TM is the target material amount, CM is the current material amount, MM is the material move amount, RM is the remaining material amount, and AM is the actual material amount.

### 3.1. Material model

PTO method adopts the modified SIMP approach [9], which is a density approach, for better search performance while maintaining near 0/1 solutions. The modified SIMP approach reads

$$E\left(\rho \right)=E_{min}+\rho^{p}E_{0}$$ (6)

where *E* is the density dependent Young’s modulus, *E*_*min*_ is a small Young’s modulus (typically 10^−9^) assigned to void elements in order to avoid obtaining a singular stiffness matrix, *E*_*0*_ is the Young’s modulus of the solid material, and *p* is the penalty coefficient (typically 3) [9]. The modified SIMP approach makes it redundant to have a lower bound for density *ρ*_*min*_ to avoid the stiffness singularities since *E*_*min*_ already serves the said purpose. The modified SIMP approach drives densities towards 0 and 1 since volume varies linearly as stiffness varies in the order of *p*.

### 3.2. Stress constraint

PTO method employs the following maximum function as a stress constraint

$$max\left\{\sigma_{i}\right\}\leq \sigma_{elastic\;limit}$$ (7)

where *σ*_*i*_ is the stress at element *i* and it is taken to be the von Mises stress at the geometric center of the element. The details of stress calculation are presented in the following. The stress constraint entails that the stress does not exceed the elastic limit at any element in the system. Therefore, the constraint provides a tight control on the stress levels owing to the maximum function. It should be noted that the maximum function is not differentiable, and thus cannot be used with sensitivity methods. Instead, sensitivity methods usually employ a *p*-norm of stress [14]. The *p*-norm stress measure is not as tight as the maximum stress measure unless the value of *p* is very big. As such, for *p = ∞*, the p-norm stress measure is equivalent to the maximum stress measure. In addition, the *p*-norm stress measure does not have a physical meaning as the maximum stress measure does [14]. Finally, implementation of the maximum stress measure is the simplest compared to the other stress measures.

### 3.3. Density filtering

The PTO method incorporates a density filtering. In the work of Bruns [45], a simple cone density filtering is introduced as the following

$$\rho_{i}=\frac{\sum \;w_{ij}d_{j}}{\sum \;w_{ij}}\;where\;w_{ij}=\{\begin{array}{cc} \frac{r_{0}-r_{ij}}{r_{0}} & for\;r_{ij}<r_{0}\\ 0 & for\;r_{ij}\geq r_{0} \end{array}$$ (8)

*ρ*_*i*_ is the filtered density of element *i*, *w*_*ij*_ is the filtering weight of elements *i* and *j*, *d*_*j*_ is the non-filtered density of element *j*, *r*_*ij*_ is the distance between elements *i* and *j*, and *r*_*0*_ is the filter radius. The weight is inversely proportional to the distance between the element and its neighbors. In this sense, the cone density filtering is actually nothing but local averaging. Besides, it preserves the volume. It should be noted that it is always filtered densities that are presented in the results section. Filtering is endorsed to have the following advantages:

(i) Small scale features such as jagged edges, narrow members, and sharp interfaces are prevented [14].
(ii) As a result of smoothing, a blurred region around the structural members is obtained [14].
(ii) The algorithm is saved from getting stuck in local minima [14].
(iv) Checkerboard phenomenon is prevented [46].
(v) Ensures existence of solution, although this is not proven yet [7].
(vi) Imposes a constraint on minimum length scale of the design [46].

As a separate note, even if the method had sensitivity, it is argued that sensitivity filtering is not suitable for the stress problem [14]. A number of filtering methods are presented by Sigmund [46]. In addition, two alternative filtering schemes for the Top88 code are introduced by Andreassen [9].

### 3.4. Boundary conditions

Finite element (FE) problem definitions are required to be accompanied with some essential and natural boundary conditions. These prescribed boundary conditions are usually concentrated and their correct imposition to the problem domain is crucial for the FE solution. In a similar manner, it is vital to correctly handle the boundary conditions for the topology optimization solution. We experienced that exclusion of the elements near the boundary conditions from the topology optimization problem actually results with different solutions from those obtained when these elements are included. Moreover, the exclusion of elements near the boundary conditions yields better optimization results, which may be misleading. On the other hand, imposing boundary conditions to only a few elements leads to poor topology optimization behavior due to compliance/stress concentration [14, 16, 47]. Consequently, the best practice is to distribute the boundary conditions to a sufficient number of elements in order to provide the topology optimization algorithm to work properly, as followed by many researchers [14, 16, 48]. If the resulting structure is suspected to be fragile for loading conditions as pointed out by Holmberg [39], more material can be added near the loading regions at the post-processing phase.

### 3.5. Stress measure

As stated earlier, von Mises stress is measured at the geometric center of the elements. In the following, only two-dimensional (2-D) examples with plane stress and bilinear square elements of length *L* are considered. The von Mises stress in 2-D is given by

$$\sigma_{vM}=\sqrt{\sigma_{x}^{2}+\sigma_{y}^{2}-\sigma_{x}\sigma_{y}+3\sigma_{xy}^{2}}$$ (9)

The stress tensor in 2-D is expressed as

$$\sigma =\left\{\begin{array}{c} \sigma_{x}\\ \sigma_{y}\\ \sigma_{xy} \end{array}\right\}$$ (10)

And obtained by

σ=DBu (11)

where ***D*** is the constitutive matrix, ***B*** is the shape function derivative matrix, and ***u*** is the displacement vector. The constitutive matrix for plane stress in 2-D is as the following

$$D=\frac{E}{1-v^{2}}\left[\begin{array}{ccc} 1 & v & 0\\ v & 1 & 0\\ 0 & 0 & \left(1-v\right)/2 \end{array}\right]$$ (12)

where *E* is the Young’s modulus and *ν* is the Poisson’s ratio. For linear shape functions for a bilinear square element in 2-D, ***B*** is given by

$$B=\frac{1}{2L}\left[\begin{array}{ccc} \begin{array}{ccc} -1 & 0 & 1\\ 0 & -1 & 0\\ -1 & -1 & -1 \end{array} & \begin{array}{ccc} 0 & 1 & 0\\ -1 & 0 & 1\\ 1 & 1 & 1 \end{array} & \begin{array}{cc} -1 & 0\\ 0 & 1\\ 1 & -1 \end{array} \end{array}\right]$$ (13)

Lastly, ***u*** is the element displacement vector represented as

$$u=\left\{\begin{array}{c} \begin{array}{c} u_{1x}\\ u_{1y}\\ u_{2x} \end{array}\\ \begin{array}{c} u_{2y}\\ u_{3x}\\ u_{3y} \end{array}\\ \begin{array}{c} u_{4x}\\ u_{4y} \end{array} \end{array}\right\}$$ (14)

The term “stress” in the results section always refers to the von Mises stress at the geometric center of the square elements.

### 3.6. MATLAB programs

**Fig 1:**
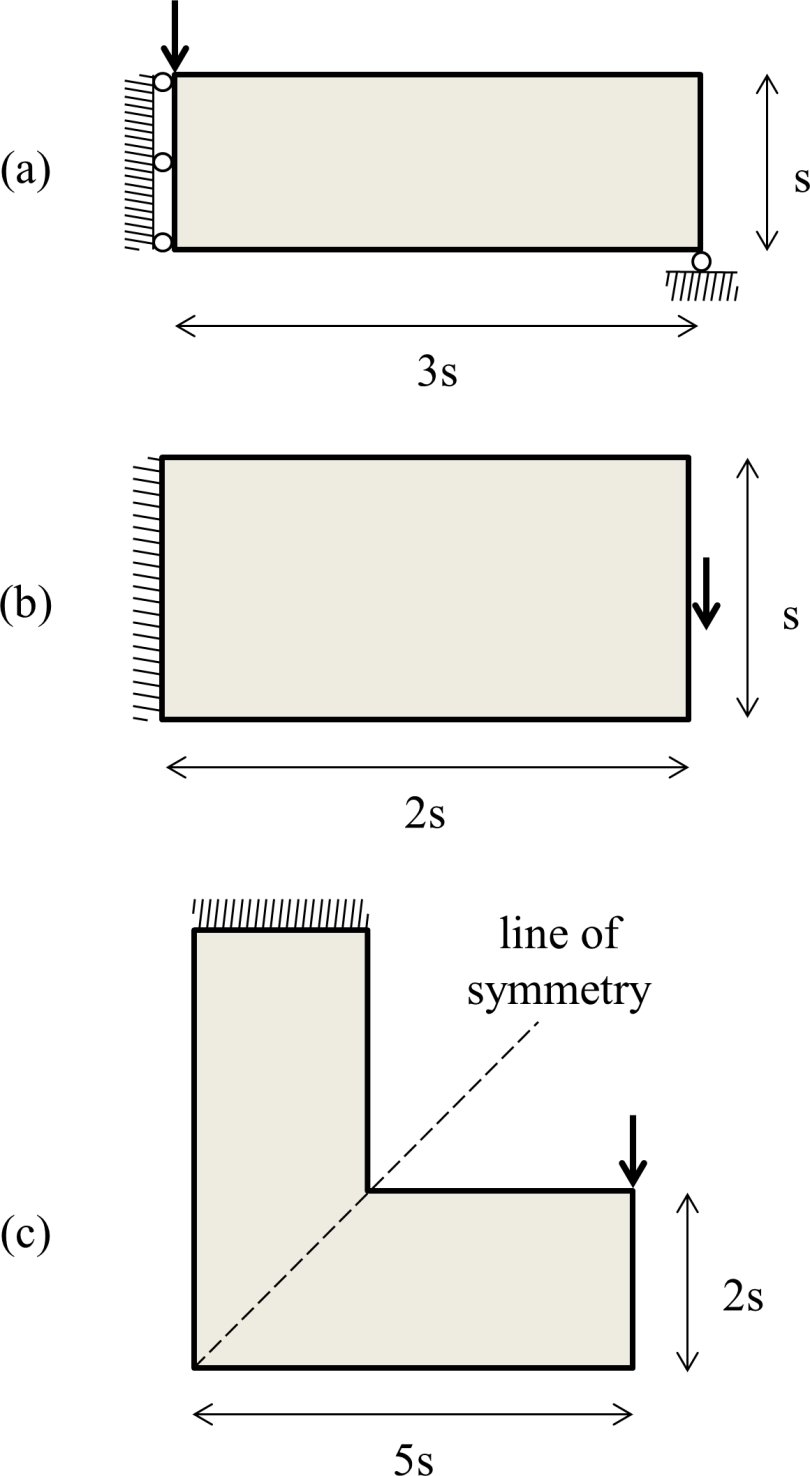
Numerical examples: (a) MBB beam–only right half (a2) of the full design domain (a1) is considered due to symmetry, (b) Cantilever beam, and (c) L bracket.

Two separate MATLAB programs that solve the stress and compliance problems for the MBB beam in bending (Fig 1A) are presented. In short, the MBB beam in bending is referred to as MBB beam hereafter. It is important to acknowledge that the computer programs substantially inherit from the 88-line MATLAB code by Andreassen et al. (Top88 hereafter), such as setup and solution of FE system. In particular, the only major modification is undertaken in optimization algorithm and some other minor modifications elsewhere. Minor modifications include addition of stress analysis and removal of sensitivity analysis. Furthermore, a few extra input parameters are introduced to control: the element edge length, number of elements the load is distributed on, and lower and upper bounds on density. The latter is introduced for different design needs as it may be asked to have a lower bound on density for a cellular structure. This intervention should not conflict with the SIMP approach as long as the penalization factor *penal* is accordingly justified.

The computer programs are cast as MATLAB functions that can be called from the MATLAB command window or other MATLAB programs. The first computer program is for the MBB beam example solved for the stress problem (S1 Appendix). In this case, the function is called as the following

$$\begin{array}{c} x=PTOs\_mbb\;(E0,Emin,L,lv,ld,nelx,nely,nu,\\ \;penal,q,rmin,vmslim,xlim) \end{array}$$

where *x* is the elemental densities, *E*_*0*_ is the Young’s modulus, *E*_*min*_ is the Young’s modulus assigned to void elements, *L* is the element edge length, *lv* is the load value, *ld* is the number of elements displacement and force loads are distributed on, *nelx* is the number of elements in *x* dimension, *nely* is the number of elements in *y* dimension, *nu* is the Poisson’s ratio, *penal* is the penalization factor in the modified SIMP formula, *q* is the proportion exponent, *rmin* is the filter radius, *vmslim* is the stress constraint limit, and *xlim* is a *1x2* vector consisting of lower and upper bounds on density, respectively.

**Fig 2:**
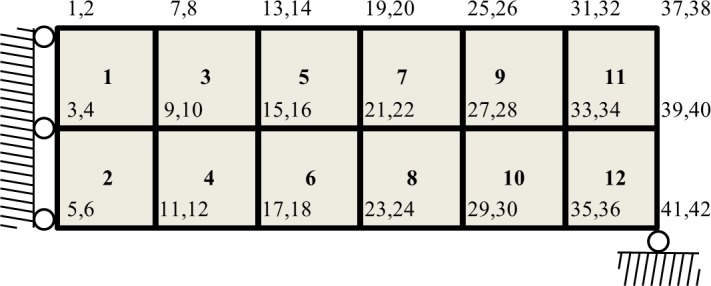
Numbering of DOF, nodes, and elements in right half of the MBB beam: starting from top-left and proceeding in column-wise order.

Lines 5–9 prepare the element stiffness matrix *KE* that is to be multiplied by the Young’s modulus *E* to get to its final form. Lines 10–12 prepare the *edofMat* matrix that is in size of *(element number) x (8)* and consists of degrees of freedoms (DOF) of each element in a row. Numbering of DOF, nodes, and elements in the system starts from top-left and proceeds in column-wise order (Fig 2).

Lines 13–14 prepare *iK* and *jK* vectors that represent the indices of nodes in the global stiffness matrix. Lines 16–19 form the force sparse vector *F* with respect to input load value *lv* and distribution parameter *ld*. Line 21 initializes the displacement vector *U* to zero. Line 22 composes the set of fixed DOFs with respect to the input load distribution parameter *ld*. Lines 23 and 24 composes the sets of all DOFs and free DOFs, respectively. The set of free DOFs *freedofs* is later employed when solving the FE system. Lines 26–27 prepare the element shape function derivative matrix *B* and constitutive matrix *DE* for stress analysis. The latter is to be multiplied by the Young’s modulus *E* to get to its final form.

Lines 29–48 build the density filter sparse matrix. In specific, lines 33 and 34 loop for every element position, and lines 36 and 37 loop for neighbor element positions. Lines 40–41 save the indices for pair of neighbors. Line 42 computes and saves the weight of density filtering for the pair of neighbors from the distance between them if the distance is smaller than the input filter radius. After exiting the loop, lines 47 and 48 create the density filter sparse matrix and normalize it, in order.

The main loop takes place between lines 53 and 90. It first carries out the FE analysis in lines 56–59, and finds the displacements *U*. More specifically, the main loop conducts FE analysis by populating the global stiffness sparse matrix *K* in lines 57–58 with the updated Young’s modulus values *E* from line 56, and then solving the FE system *KU = f* in line 59. The main loop follows by the stress and compliance analyses. Stress analysis computes the elemental stress tensors in line 61 and the elemental equivalent von Mises stresses in line 62. Compliance analysis computes the elemental compliances into a vector in line 64 and reshapes this vector into a matrix by the corresponding number of elements in each dimension in line 65. The main loop prints out the results to the command window in lines 67–68; and, plots the elemental densities and stresses normalized by the maximum value of corresponding matrices in lines 70–72.

In line 74, the main loop checks for the termination criteria, that is whether the maximum elemental stress in the system is close to the stress constraint limit within a tolerance (i.e., 0.001) and number of iterations is more than 50. The latter is introduced to inhibit immature terminations, which occurred only one time in authors’ experience. If the termination criterion returns true, the main loop exits, and simulation ends.

Lines 76–89 consist of the core PTOs algorithm. Initially, lines 76–80 determine the target material amount with respect to the maximum elemental stress in the system. In that, if the maximum elemental stress exceeds the stress constraint, more material is added, or removed otherwise. The added/removed material amount is equal to the multiplication of the total number of elements by 0.001. Following, lines 84–89 represents the inner loop that iteratively distributes the target material amount proportionally to the elemental stress values. This proportion is computed out of the loop in line 83 for efficiency. The proportion is extended by the proportion exponent *q*. The inner loop starts with distribution of the remaining material in line 85. Then, lines 86 and 87 filter the distributed material and enforce density limits on the elemental densities, respectively. The inner loop ends with computation of remaining material amount in line 88. The inner loop terminates when the remaining material amount is less than or equal to 0.001, as checked in line 84.

The second computer program is for the MBB beam example solved for the compliance problem (S2 Appendix). In this case, the function is called as the following

$$\begin{array}{c} x=PTOc\_mbb\;(alpha,E0,Emin,L,lv,ld,nelx,nely,\\ \;nu,penal,rmin,vmslim,xlim) \end{array}$$

where *alpha* (i.e., *α*) is the history coefficient and other arguments are identical to PTOs, except that the proportion exponent *q* is omitted (the reason will be detailed in section 3.7). Although the lines of PTOs and PTOc do not match at the same line number all the time, the flow and steps of the programs are largely the same. The differences are detailed in the following.

PTOc has a new variable that first appears in line 51, named *xNew*, and stores the optimized elemental densities in the current iteration of the loop. Later, line 88 updates elemental densities *x* with respect to the history coefficient *alpha* as a linear combination of elemental densities from the previous (i.e., *x*) and current (i.e., *xNew*) iterations. Line 76 checks whether the termination criteria is satisfied. That is, if the change in the maximum elemental densities between two successive iterations (this *change* is computed in line 89) is smaller than 0.01 and the number of iterations is more than 50. The former criterion is different than that of PTOs since PTOc satisfies the volume constraint a priori in line 78 as will be explained later. In contrast, PTOs searches for a distribution until the stress constraint is satisfied, hence a posteriori.

Line 78 computes the target material amount as dictated by the input constraint on total volume fraction *vlim*. This value is constant during the course of the simulation. As can be followed from lines 81 and 83, PTOc distributes the material amount in proportion to the elemental compliance values. The proportion is more direct (and linear) compared to PTOs since there is no use of proportion exponent.

In case the above descriptions of computer programs are not clear enough, the reader is referred to two other MATLAB codes and corresponding papers, namely 99-line code [7] and 88-line code [9], for alternative descriptions due to the fact that current codes mainly inherit from the two referred codes.

The computer programs are highly flexible and extensible. For instance, the programs can easily be modified to insert a prescribed void or solid region in the design by constraining the corresponding elemental densities to 0 or 1 in the inner loop right after updating *x* in line 87 in PTOs and 85 in PTOc. For another instance, PTOs can be extended to minimize volume fraction under both stress and compliance constraints. Then, in addition to the check for elemental stresses, the same practices should be implemented for elemental compliances. This way, material should be added to the system when either of the constraints is not satisfied, and material should be removed from the system when both constraints are satisfied. In like manner, the simulation should terminate when both constraints are satisfied at the same time.

The computer programs are unitless. However, a set of units can be attached to attain a physical relevance. A set of consistent units are kg for mass, meter for length, and second for time. Then, force units are Newton, stress units are Pa, and compliance units are Nm. An alternative set of consistent units are ton for mass, mm for length, and second for time. Then, force units are Newton, stress units are MPa, and compliance units are Nmm. It should be carefully noted that *ld*, *nelx*, *nely*, and *rmin* are in units of element, regardless of the element edge length *L*. That is, an *ld* value of 3 means that load is distributed on 3 elements. Also, the element edge length *L* and thickness of the elements are considered to be unity. As a result, the volume of the elements is unity; therefore, it is eliminated from the computer programs. Additionally, *xlim* and *vlim* have normalized values between 0 and 1. That is, a *vlim* value of 0.5 means that 50% of the material amount of a full solid design *(number of elements in x)* x *(number of elements in y)* is to be filled in.

The computer programs are verified against the ANSYS commercial FE software by means of comparing displacement, compliance, and stress values. It is noteworthy that the stress values presented in this work and by the computer programs are actual stress values meaning that they are not normalized, multiplied by density, or norms of actual stresses values.

### 3.7. Control parameters

Two control parameters are defined to fine tune the behavior of the PTO algorithm: proportion exponent (q) and history coefficient (α). The proportion exponent controls the degree of proportion between the elemental density value and elemental stress or compliance values for the stress and compliance problems, respectively. For instance, a quadratic proportion for the stress problem means that the total material amount is distributed to elements in proportion to the square of the elemental stress values. The other control parameter is the history coefficient. It controls the ratio of dependence of elemental density to its older value from the previous iteration. For instance, a value of 0.5 means that the elemental densities are blended such that half of their new values come from the previous iteration and the other half come from the optimized values in the current iteration.

In order to increase the accuracy and efficiency of the algorithms, a parametric study of the proportion exponent and history coefficient for PTOc and PTOs are carried out using the MBB beam example. PTOc and PTOs are called with the following arguments: 1 for Young’s modulus *E*_*0*_, 0.3 for Poisson’s ratio *ν*, 10^−9^ for Young’s modulus assigned to void regions *E*_*min*_, 3 for penalty value for modified SIMP approach *penal*, 1 for load value *lv*, 3 for number of load imposed elements *ld*, 120 for number of elements in *x*-direction *nelx*, 40 for number of elements in *y*-direction *nely*, 0 and 1 for lower and upper elemental density bounds *xlim*, 0.35 for volume constraint *vlim* in PTOc, 1.08 for stress constraint *vmslim* in PTOs, 1 for element edge length *L*, and 1.5 for filter radius *r*_*min*_. Both algorithms are tested for history coefficients from 0.0 to 0.9 in increments of 0.1 and for proportion exponents from 0.25 to 3.0 in increments of 0.25. The results for PTOc are listed in Table 3 where non-bold font results are eliminated due to high compliance and/or iteration number. Among the bold font results, values of 0.5 for the history coefficient and 1 for the proportion exponent are reasonable settings for having low compliance and iteration number and being away from unstable values. Although the results obtained by a value of 0.9 for the history coefficient are also good, they are discarded because of their strong dependency on the history as this relation may be troublesome for the untested cases. As a result, the proportion exponent has no effect in the PTOc algorithm and thus omitted from the implementation. The results for PTOs are listed in Table 4 where non-bold font results are eliminated due to high volume and/or iteration number. Among the bold font results, values of 0 for the history coefficient and 2 for the proportion exponent is the best setting for having the lowest volume. As a result, the history coefficient has no effect in the PTOs algorithm and thus omitted from the implementation. In spite of the suggested settings, it is encouraged to experiment with the parameters to better utilize the method in other specific cases.

**Table 3:** Parametric results for the PTOc: Compliances (iteration number) are given for *α* from 0 to 0.9 and *q* from 0.25 to 3.

q\α	0	0.1	0.2	0.3	0.4	0.5	0.6	0.7	0.8	0.9
**0.25**	6.4x10^9^	1248.48	1248.48	1248.48	1248.48	1248.49	1248.53	1248.88	1251.6	1277.09
	(1000)	(51)	(51)	(51)	(51)	(51)	(51)	(51)	(51)	(51)
**0.5**	6.4x10^9^	2165.83	303.52	299.97	300.44	300.99	301.87	341.87	623.59	703.63
	(1000)	(1000)	(132)	(145)	(165)	(195)	(240)	(214)	(55)	(51)
**0.75**			331.72	267.29	**268.33**	268.55	268.9	**274.05**	273.89	**278.52**
			(1000)	(1000)	**(165)**	(193)	(201)	**(151)**	(213)	**(185)**
**1**			2594.4	265.87	**265.12**	**266.61**	**266.25**	266.36	266.56	**272.1**
			(1000)	(1000)	**(164)**	**(170)**	**(181)**	(200)	(221)	**(134)**
**1.25**					264.89	264.11	264.23	264.84	265.35	266.36
					(188)	(223)	(265)	(250)	(231)	(206)
**1.5**					319.41	263.88	264.47	264.41	264.48	265.62
					(1000)	(196)	(286)	(279)	(244)	(191)
**1.75**					325.07	263.71	263.58	264.27	264.64	265.12
					(1000)	(583)	(378)	(233)	(213)	(203)
**2**						262.11	262.39	263.87	264.25	**265.15**
						(1000)	(304)	(245)	(212)	**(150)**
**2.25**						348.2	262.69	263.66	**264.47**	**265.05**
						(1000)	(489)	(254)	**(163)**	**(156)**
**2.5**							267.57	263.55	264.1	**264.95**
							(1000)	(303)	(195)	**(159)**
**2.75**							271.25	263.27	263.93	**264.9**
							(1000)	(338)	(276)	**(169)**
**3**								262.08	263.57	264.87
								(575)	(288)	(188)

Non-bold results have 10% more compliance and/or iteration than the result in the box. Non-convergent simulations are terminated at the 1000^th^ iteration. The dashed cases are assumed to be non-convergent as is the case for similar settings.

**Table 4:** Parametric results for the PTOs: Volume (iteration number) are given for *α* from 0 to 0.9 and *q* from 0.25 to 3.

q\α	0	0.1	0.2	0.3	0.4	0.5	0.6	0.7	0.8	0.9
**0.25**	0.60	0.60	0.60	0.60	0.60	0.60	0.60	0.60	0.60	0.59
	(111)	(121)	(137)	(155)	(182)	(218)	(271)	(362)	(542)	(1000)
**0.50**	0.39	0.39	0.39	0.39	0.39	0.39	0.39	0.39	0.51	0.51
	(135)	(149)	(168)	(192)	(224)	(268)	(334)	(443)	(67)	(133)
**0.75**	0.39	0.39	0.39	0.39	0.39	0.39	0.39	0.39	0.39	0.41
	(121)	(135)	(152)	(172)	(203)	(243)	(303)	(404)	(601)	(1000)
**1.00**	0.38	0.39	0.39	0.39	0.39	0.39	0.39	0.39	0.39	0.41
	(125)	(133)	(159)	(183)	(212)	(242)	(300)	(397)	(594)	(1000)
**1.25**	0.36	0.39	0.39	0.39	0.39	0.39	0.39	0.39	0.39	0.40
	(164)	(128)	(143)	(172)	(206)	(241)	(297)	(405)	(590)	(1000)
**1.50**	0.38	0.39	0.37	0.38	0.35	0.39	0.39	0.39	0.39	0.40
	(138)	(140)	(208)	(184)	(253)	(254)	(314)	(395)	(586)	(1000)
**1.75**	**0.32**	0.36	0.36	0.36	0.38	0.38	0.36	0.39	0.39	0.40
	**(197)**	(179)	(209)	(216)	(209)	(252)	(365)	(399)	(584)	(1000)
**2.00**	**0.31**	0.30	0.36	0.39	0.36	0.39	0.34	0.39	0.38	0.50
	**(206)**	(248)	(200)	(180)	(251)	(246)	(415)	(416)	(619)	(81)
**2.25**	0.35	0.34	0.36	0.36	0.37	0.38	0.39	0.36	0.40	0.49
	(170)	(205)	(209)	(224)	(246)	(255)	(306)	(487)	(584)	(95)
**2.50**	0.28	0.25	0.34	0.37	0.39	0.38	0.49	0.49	0.39	0.50
	(243)	(325)	(237)	(211)	(228)	(261)	(56)	(63)	(638)	(87)
**2.75**	**0.32**	0.31	0.32	0.35	0.37	0.36	0.39	0.35	0.34	0.50
	**(200)**	(240)	(255)	(245)	(254)	(307)	(324)	(546)	(889)	(83)
**3.00**	**0.32**	0.31	0.33	0.33	0.37	0.34	0.37	0.36	0.45	0.50
	**(202)**	(341)	(237)	(262)	(256)	(348)	(352)	(506)	(334)	(58)

Non-bold results have 10% more compliance and/or iteration than the result in the box. Non-convergent simulations are terminated at the 1000^th^ iteration.

## Numerical Examples

Results section consists of three parts. The first part shows that PTOs and PTOc work well for topology optimization. The second part compares PTOc to Top88, and the third compares PTOs to PTOc. In all parts, three numerical examples that are defined in Fig 1 are considered.

In all three examples, material properties are input as 1 for Young’s modulus *E*_*0*_, 0.3 for Poisson’s ratio *ν*, and 10^−9^ for Young’s modulus assigned to void regions *E*_*min*_. Penalty value for modified SIMP approach *penal* is set to 3. A load value of 1 (*lv*) is imposed over 3 elements (*ld*). Lower and upper bounds *xlim* on elemental density are limited to 0 and 1. Element edge length *L* and filter radius *rmin* are set to 1 and 1.5, respectively. Thickness of the domain is assumed to be equal to 1. As stated earlier, *q* is tuned to 2 for PTOs and *α* is tuned to 0.5 for PTOc.

In the first example, right half of the MBB beam is discretized by 120x40 (*nelx* x *nely*) elements. The beam is fixed in *x*-dimension on the left edge due to symmetry and fixed in *y*-dimension on the lower-right corner. A normal force is applied on the upper-left corner. In the second example, the cantilever beam is discretized by 120x60 (*nelx* x *nely*) elements. The beam is fixed in both *x* and *y*-dimensions on the left edge and a shear force is applied at the middle of the right edge. In the third example, the L bracket is discretized by 100x40 (*nell* x *nels*) elements in long (*l*) and short (*s*) edges. The bracket is fixed in both *x* and *y*-dimensions on the upper edge and a normal force is applied on the top of the most right edge.

### 4.1. Validation of PTOc and PTOs

The first part of the results section runs PTOc and PTOs for the three examples. Initially, PTOc is run for a volume fraction 0.35 and then the output stress value is input to the PTOs as a constraint. For instance, PTOc is called to solve the MBB example by the following command

PTOc_mbb(0.5,1,1e−9,1,1,3,120,40,0.3,3,1.5,0.35,[0,1])

The simulation ends with a stress 1.08. Then, PTOs is called with this stress value by the following command

PTOs_mbb(1,1e−9,1,1,3,120,40,0.3,3,2,1.5,1.08,[0,1])

**Fig 3:**
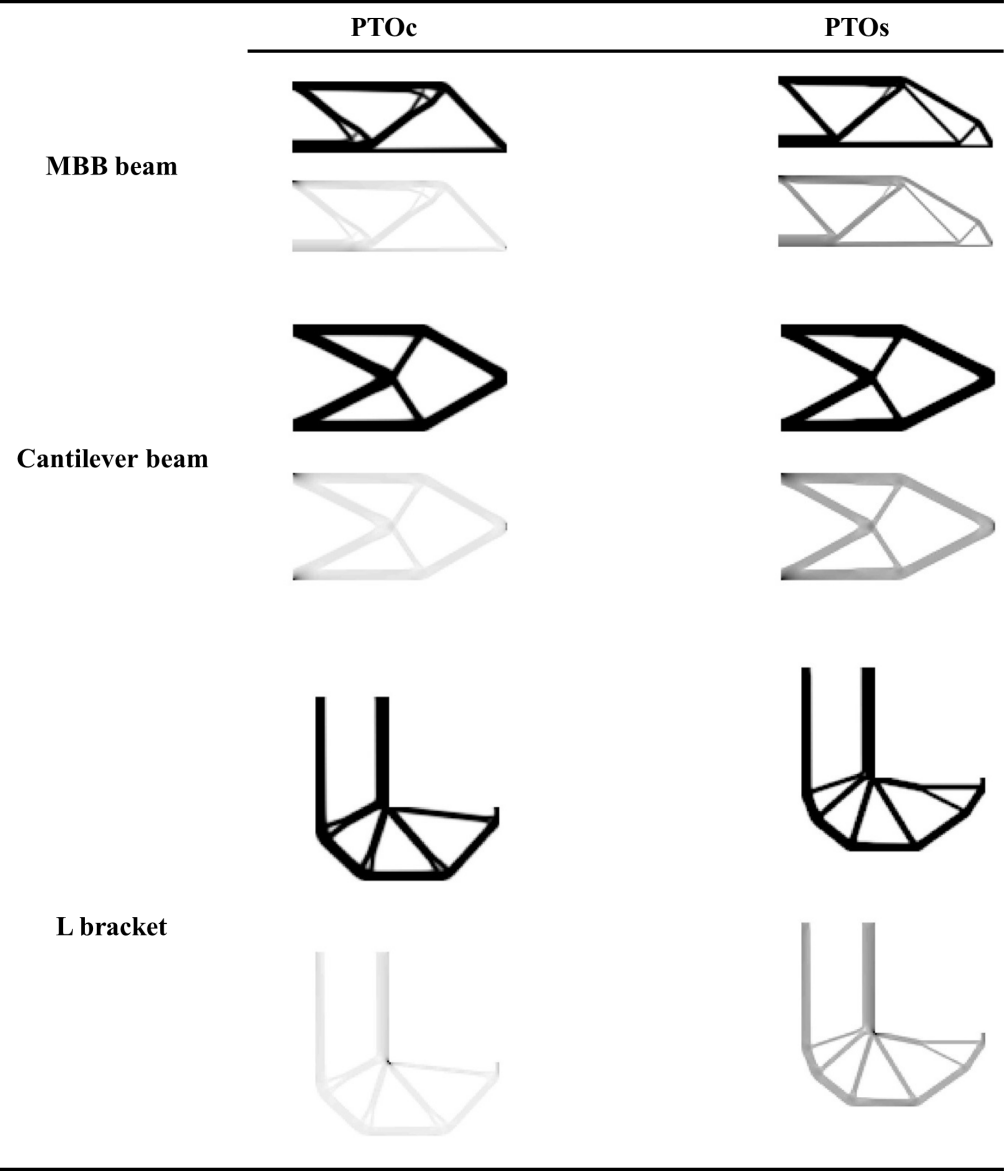
Topologies and compliance (PTOc) or stress (PTOs) distributions obtained from the MBB beam, cantilever beam, and L bracket examples.

This routine is repeated for the cantilever beam and L bracket examples. The simulations converge with the results tabulated in Table 5 to the topologies shown in Fig 3. Some remarks are in order.

**Table 5:** Number of iterations, volume fraction, compliance, maximum stress, and contrast index obtained from MBB beam, cantilever beam, and L bracket solved by PTOc and PTOs.

		Number of iterations	Volume fraction	Compliance	Max stress	Contrast index
**MBB beam**	**PTOc**	170	0.35	266.61	1.08	0.80
**MBB beam**	**PTOs**	206	0.31	294.92	1.08	0.83
**Cantilever beam**	**PTOc**	106	0.35	88.54	0.57	0.85
**Cantilever beam**	**PTOs**	164	0.34	90.62	0.57	0.88
**L bracket**	**PTOc**	78	0.35	235.25	1.05	0.83
**L bracket**	**PTOs**	187	0.33	248.97	1.05	0.85

All six cases show that for the same stress level, PTOs results with higher compliance but lower volume fraction. On average, PTOc solutions have 6.3% less compliance; and, PTOs solutions have 7.3% less volume. The topologies are almost identical for the cantilever beam example, but they are considerably different for the MBB beam and L bracket examples. PTOc tends to have thicker structural members while PTOs inclines towards more number of structural members. The contrasts of topologies are investigated by an index defined as

$$\text{Contrast index}=\frac{NoE\;\text{with}\;\rho_{i}<0.01\;\text{or}\;\rho_{i}>0.99}{\text{Total}\;NoE}$$ (15)

where *NoE* is the number of elements and *ρ*_*i*_ is the elemental density. The results are given in Table 5. On average, PTOc and PTOs topologies result with 0.83 and 0.85 contrast indices, respectively. The contrast indices indicate that both PTOs and PTOc provide with near black-and-white solutions.

The user has a few options to get completely black-and-white solutions at the end of the simulation. Among these are continuation methods that suggests progressive decrease of the filter radius [19] or increase of the SIMP penalization factor [18] during the course of the simulation. Another option is to use post-processing tools, such as projection schemes, to drive the simulation result to a black-and-white final result [18]. These methods are considered to be efficient and effective, but partially heuristic.

### 4.2. Comparison of PTOc to Top88

**Fig 4:**
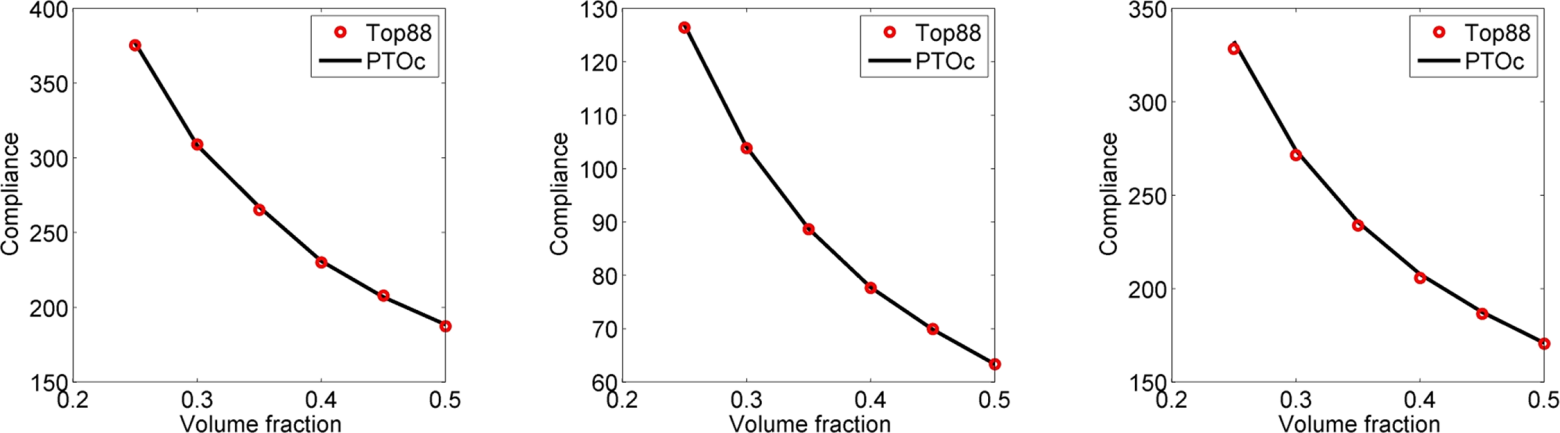
Comparison of compliance versus volume fraction curves of PTOc and Top88 for the MBB beam (left), cantilever beam (center), and L bracket (right) examples.

The second part of results section compares PTOc to Top88 for the three examples. It should be clarified that the original Top88 code is only for the MBB beam example; but, it has been extended to solve the cantilever beam and L bracket examples. In this connection, Top88 represents an OC method with sensitivities. For each example, both programs are called by a set of identical inputs. For instance, material properties and penalization factor, density filter and its radius, and loading value and distribution are set the same. As a result, simulations for each method are identical except the optimization algorithms. PTOc and Top88 are run for a number of volume fractions *vlim* from 0.25 to 0.50 in increments of 0.05. Fig 4 shows comparison of compliances for the three examples. The figures demonstrate that the compliance versus volume fraction curves of PTOc and Top88 are indistinguishable for all three examples. Also, average number of iterations and simulation times are compared in Table 6. As can be seen, Top88 performs much better for the MBB beam example and slightly better for the cantilever beam example. On the other hand, PTOc performs much better for the L bracket example. On average, Top88 algorithm performs slightly better by 4.0% in iteration numbers and 6.3% in simulation times. The conclusion is that none of the methods is superior to the other in terms of efficiency in general but they have varying performances depending on the example.

**Table 6:** Comparison of iteration numbers and simulation times of Top88 and PTOc.

		Top88	PTOc	Relative comparison100x(1-PTOc/Top88)
**MBB Beam**	**Iteration number**	233.0	308.5	-32.4
**MBB Beam**	**Time (s)**	36.19	50.34	-39.1
**Cantilever Beam**	**Iteration number**	150.0	153.2	-2.1
**Cantilever Beam**	**Time (s)**	35.46	36.89	-4.0
**L Bracket**	**Iteration number**	201.0	155.5	22.6
**L Bracket**	**Time (s)**	42.93	32.50	24.3
**Average**	**Iteration number**	194.7	205.7	-4.0
**Average**	**Time (s)**	38.19	39.91	-6.3

**Fig 5:**
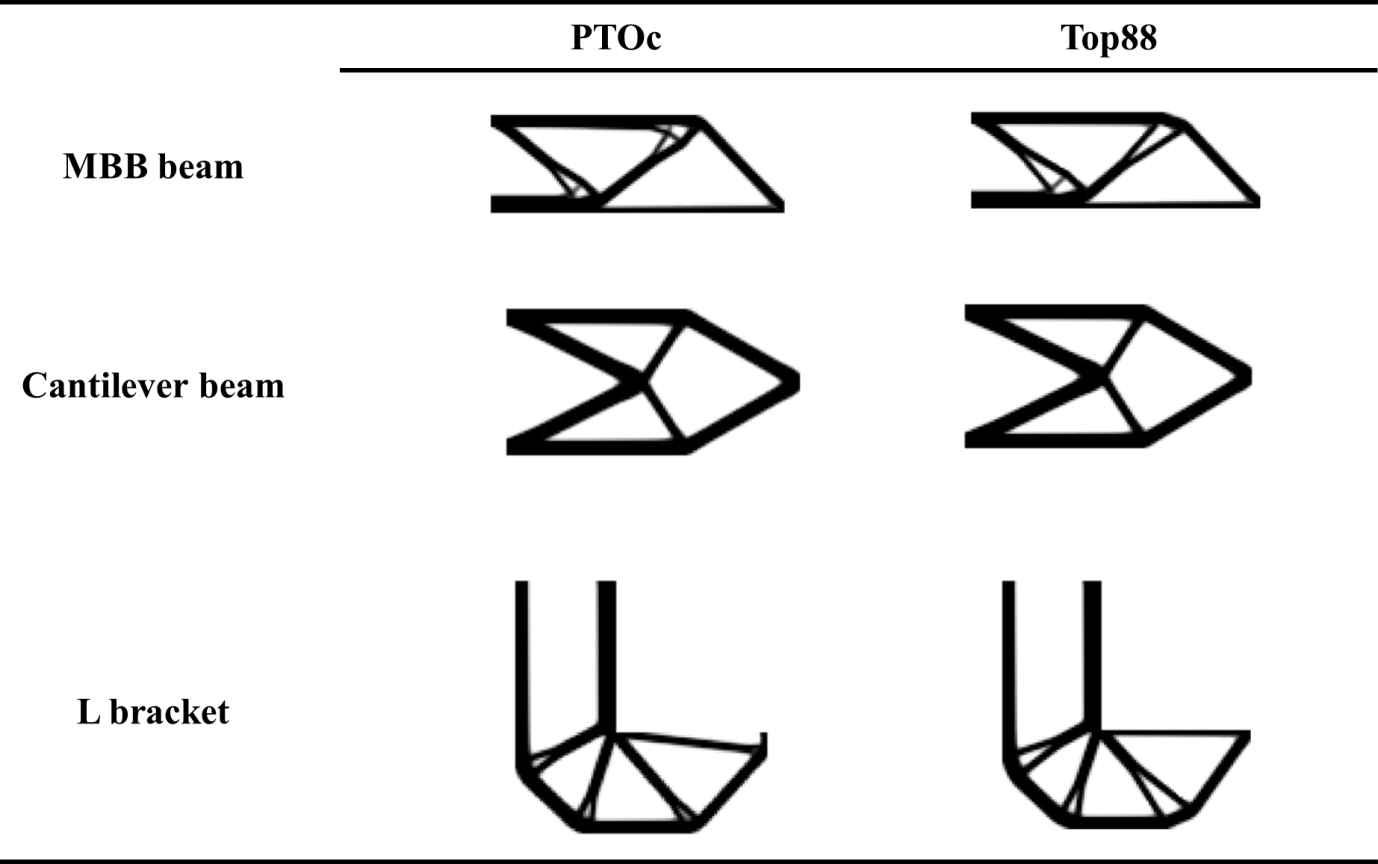
Comparison of topologies of PTOc and Top88 for the MBB beam, cantilever beam, and L bracket examples.

Fig 5 compares topologies obtained by running PTOc and Top88 for a volume fraction 0.35 for three examples. Topologies are similar for the cantilever beam example and remarkably different for the MBB beam and L bracket examples. The most prominent difference is the tiny feature near the loading in the L bracket topology solved by PTOc. Such a tiny feature is not a good design practice since it is fragile against loadings in traverse directions. Thus, these kinds of considerations should be made by the designer in the post-processing phase. The topologies are also compared by their contrast indices. Contrast indices for Top88 topologies are 0.81, 0.86, and 0.83 for the examples in the presented order. Compared to contrast indices of PTOc in Table 5, contrast indices between the two methods are not different more than 0.01.

### 4.3. Comparison of PTOs to PTOc

**Fig 6:**
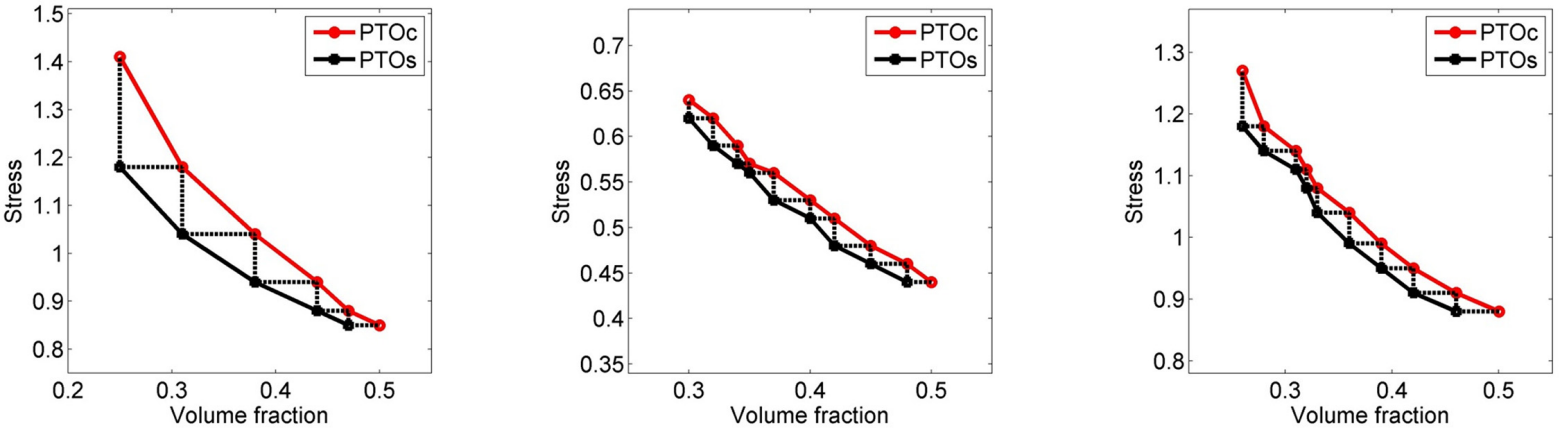
Comparison of stress versus volume fraction curves of PTOs and PTOc for the MBB beam (left), cantilever beam (center), and L bracket (right) examples. Dashed lines indicate the links between PTOs and PTOc. A horizontal dashed line means stress output of PTOc is input to the PTOs and a vertical dashed line means volume fraction output of PTOs is input to PTOc.

The third part of results section compares PTOs to PTOc. The comparison is conducted iteratively starting from PTOc at 0.5 volume fraction. The output stress of PTOc is then input to the PTOs. Following, the output volume fraction of PTOs is input back to the PTOc, and so on. Fig 6 shows the results for MBB beam, cantilever beam, and L bracket examples. The figures show that PTOs performs better than PTOc by means of providing less volume fraction for the same level of stress and less stress for the same level of volume fraction for all three examples. This improvement is more pronounced in the MBB beam example compared to other two examples. The results are also quantified by taking the average improvements for each example, see Table 7. The results prove that the extent of improvements depend on the example. On average, though, PTOs provides 8.4% less stress for the same level of volume fraction and 5.9% less volume fraction for the same level of stress.

**Table 7:** Quantitative comparison of stress and compliance for PTOs and PTOc.

	PTOs improvement of stress (%)	PTOs improvement of volume fraction (%)
**MBB beam**	12.8	9.5
**Cantilever beam**	5.5	4.1
**L bracket**	7.0	4.0
**Average**	8.4	5.9

## Conclusions

A new topology optimization method, named PTO, is introduced. It is a non-sensitivity method, and thus eliminates difficulties emerged from analytical derivations and computational implementation of sensitivities. The achieved balance comes with a price of weaker mathematical rigor but worthy simplicity at the same time. The method possesses considerable efficiency and accuracy considering its simplicity. Even more, various comparisons to results generated by the Top88 code show that PTOc attains very similar results without use of sensitivities while maintaining same level of efficiency. On the other hand, although it is not presented here, PTOs has always been thought to be not as efficient and accurate as the state of the art methods of topology optimization field that solves stress problems for continua, especially the ones utilizing sensitivities. A comparison is left for future work.

PTO can be useful especially in educational and industrial purposes owing to its simplicity. As pointed out by Rozvany [19], industrial practitioners tend to work with methods that are easier to understand and manipulate. Naturally, students and newcomers to the topology optimization field share alike manners [7]. The method can also be useful in research due to its flexibility and extensibility. For the above purposes, two computer programs that solve the MBB beam example for stress and compliance problems are presented. The programs are individually coded in MATLAB as standalone functions and they are publicly shared in the website www.ptomethod.org. The website will be maintained with new versions, publications, extensions, and other up-to-date information.

There is more room to investigate and enhance the method, but they are left for future work. First of all, a more comprehensive parametric work is required to utilize the method at its best. Second, mesh dependency of the method is to be investigated more carefully. It is argued that filtering leads to mesh independent solutions, but this point of view is only supported by comparison of topologies [9]. The authors believe that quantitative comparisons should be carried out alongside. Third, it should be investigated whether the method would benefit from clustering of elements so that the constraints could be imposed on these clusters. It was shown that clustering of elements yield more efficient results [14]. In the current work, the method considers only one cluster that includes the whole domain. The listed future works are subject to ongoing research and will be presented in an upcoming paper.

## Supporting Information

S1 Appendix Proportional Topology Optimization stress (PTOs)—Half MBB Beam—(2015). (M)

S2 Appendix Proportional Topology Optimization compliance (PTOc)—Half MBB beam—(2015). (M)
